# Sudden bilateral hearing loss after vestibular‐evoked myogenic potentials

**DOI:** 10.1002/ccr3.5025

**Published:** 2021-11-22

**Authors:** Shinnosuke Asakura, Teru Kamogashira

**Affiliations:** ^1^ Department of Clinical Examination JR Tokyo General Hospital Shibuya‐ku Japan; ^2^ Department of Otolaryngology JR Tokyo General Hospital Shibuya‐ku Japan; ^3^ Department of Otolaryngology and Head and Neck Surgery, Faculty of Medicine University of Tokyo Bunkyo‐ku Tokyo Japan

**Keywords:** bilateral sensorineural hearing loss, noise‐induced hearing loss, vestibular‐evoked myogenic potentials

## Abstract

This case report presents bilateral hearing loss after vestibular‐evoked myogenic potential (VEMP) testing. The loud acoustic stimulus used in VEMP testing can cause noise‐induced hearing loss in some cases with high sensitivity to noise exposure or by exceeding individual cochlear safety thresholds.

## Graphical abstract

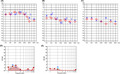

## 1 INTRODUCTION

The vestibular‐evoked myogenic potential (VEMP) is widely used in clinical practice as an effective test for evaluating the function of the saccule and the utricle. However, the acoustic stimulus used in VEMP testing is a loud sound of around 120–130 dBSPL, and the safety evaluation of the sound pressure level is still under investigation.^1^ Although only one case of bilateral hearing loss after VEMP testing has been previously reported in the literature,^2^ there is a potential risk of bilateral permanent noise‐induced hearing loss in VEMP sound stimulation.

## 2 CASE REPORT

A 78‐year‐old man with a history of lumbar spinal canal stenosis and chronic pain after lumbar spine surgery was referred to our hospital for vestibular evaluation of floating dizziness and wobbling throughout the day and a tendency to waver to the right during walking for the previous one month. He also had non‐pulsatile mild right occipital pain and stiffness in his shoulders. His medication included ketoprofen patch, fentanyl patch, fexofenadine, tramadol hydrochloride, mirogabalin besylate, mecobalamin, and limaprost alfadex, which had been newly prescribed 5 days before the vestibular examination. In the vestibular assessment, there was no gaze‐evoked nystagmus or positional nystagmus. Pure tone audiometry (PTA) showed age‐appropriate thresholds. The total trajectory lengths of posturography for 60 s were 254.17 cm (eyes open) and 339.82 cm (eyes closed), and the gravitational chart was teardrop‐shaped, suggesting a psychogenic vertigo. Videonystagmography (VNG) revealed no gaze‐evoked nystagmus, normal smooth pursuit, and good optokinetic nystagmus. The maximum slow phase velocities recorded in the ice water caloric test were 36 degrees/second on the right and 38 degrees/second on the left. The results of cervical VEMP (cVEMP) and ocular VEMP (oVEMP) testing were all normal, except for absent oVEMPs with stimulation at 1kHz on the left. The Dizziness Handicap Inventory (DHI) score was 18, the hospital anxiety and depression scale (HADS) scores were 10 (A) and 16 (D), and the Geriatric Depression Scale (GDS) score was 13. There was no evident peripheral or central vestibular dysfunction during the examination, and the patient was diagnosed with psychogenic vertigo.

**FIGURE 1:**
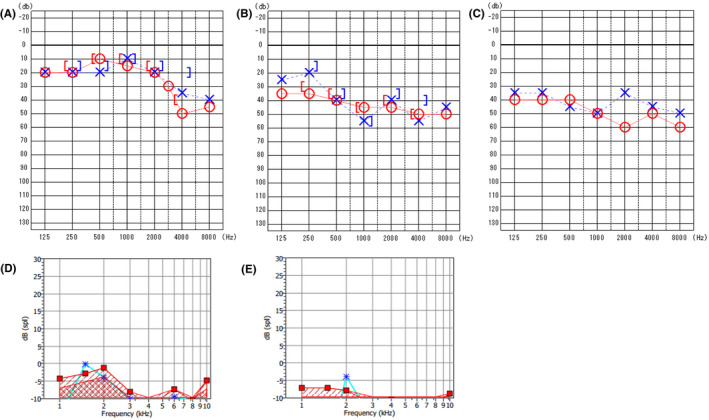
Audiograms for pre‐examination (A), post‐examination (B), and 1 month after examination (C). The hearing thresholds increased bilaterally between 500 Hz and 6000 Hz. DP‐grams after examination for (D:left, E:right)

While testing the right ear for cVEMPs with a 500 Hz stimulation, the patient suddenly stopped responding to the examiner's instructions and complained of sudden bilateral hearing loss. The examination was immediately stopped, and the physician checked both ears, but found no abnormalities. After a 5‐min break, the examination of cVEMPs and oVEMPs was continued with the patient's consent. After all tests were completed, he still complained of difficulty hearing, and pure tone audiometry showed threshold increases of about 30 dB in both ears. The results of distortion product otoacoustic emissions (DPOAE) showed no responses bilaterally. Subsequently, he was treated with oral steroid therapy and bilateral intratympanic dexamethasone, but there was no recovery in hearing thresholds after 1 month (Figure 1).

## 3 DISCUSSION

**FIGURE 2:**
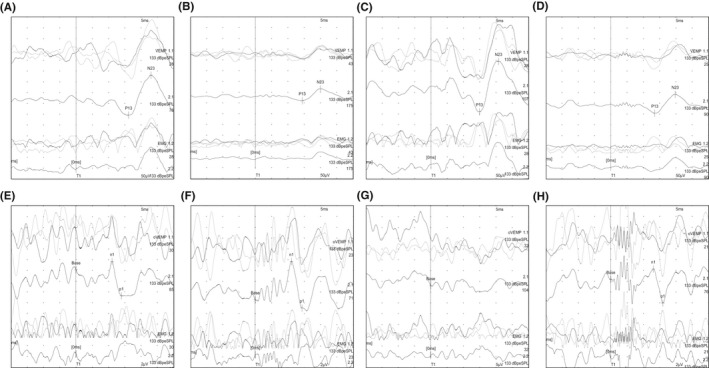
Waveforms of cVEMP (500 Hz (A: left, B: right), 1 kHz (C: left, D: right)) and oVEMP (500 Hz (E: left, F: right), 1 kHz (G: left, H: right)). Nicolet EDX (Natus) was used to record the VEMP, and the stimulation sounds were a tone burst of 500 Hz at 133 dBpeSPL (rise, 2 ms; plateau, 4 ms; and fall, 2 ms) or a tone burst of 1kHz at 133 dBpeSPL (rise, 1 ms; plateau, 2 ms; and fall, 1 ms). The numbers of stimuli for the left or right ears, respectively, were 78/175 (500 Hz) and 107/90 (1 kHz) for cVEMP and 85/71 (500 Hz) and 104/76 (1 kHz) for oVEMP. The results of cVEMP testing were normal with no significant difference in asymmetry ratio (AR) (12% (500 Hz) and 5% (1 kHz), respectively). The result of oVEMP testing with 500 Hz stimulation was normal with no difference in AR (12%); however, the oVEMP with 1 kHz stimulation was absent on the left side

There are three possible causes of bilateral hearing loss in this case. First is bilateral sudden hearing loss. Although sudden deafness usually occurs on one side only, there have been several reports of bilateral sudden deafness in the past. The reported time of onset of these cases varied from morning to noon and night, allowing the possibility that bilateral sudden deafness occurred coincidentally during the examination. The second possible cause is an enlarged vestibular aqueduct (EVA), which can cause hearing loss as a result of loud sounds. We could not evaluate the presence of EVA in our patient, however, because he did not agree to undergo MRI or CT scan. Because the onset of hearing loss with EVA generally ranges from around 10 years old up to patients in their 20s,^3^ the possibility of EVA in our elderly patient is low. The third possibility is noise‐induced hearing loss caused by VEMP stimulus sound. Although the total stimulation sound levels used in this case were within the noise and tolerance standards recommended by the Japan Society for Occupational Health, the eight‐hour equivalent continuous A‐weighted sound pressure level (L_Aeq,8hr_) of National Institutes of Occupational Safety and Health (NIOSH) was 89.1 dBA (left) and 89.5 dBA (right), which slightly exceeded the recommendations by 4.1 dB and 4.5 dB, respectively. In the past, only one case of bilateral hearing loss after VEMP testing has been reported, in which the L_Aeq,8hr_ equivalents of NIOSH were exceeded by 6 dB and 7 dB.^2^ Krause et al.^4^ reported that 27% of subjects exposed to VEMP sound stimulation (133 dBSPL) experienced auditory symptoms after VEMP testing, which had improved the next day. There were no PTA threshold increases reported (Figure 2), and DPOAE levels decreased but recovered in 24 h. Strömberg et al.^5^ also reported a DPOAE level decrease and no PTA threshold increase after VEMP sound stimulation (130 dBpeSPL). A recent study evaluating the otoacoustic emissions (OAE) and PTA in young adults undergoing VEMP testing (125 dBpeSPL) showed no significant changes.^6^ These studies show that the acoustic stimulation of VEMP can temporarily affect DPOAE levels but not PTA thresholds in young subjects depending on the stimulation sound level, although the effect in the elderly is still unknown and requires investigation. Initial guidelines for VEMP testing recommended a maximum sound pressure of 140 dBpSPL, including attention to L_Aeq,8hr_ ^7^; however, the recommended maximum safety level was reduced to 126 dBpSPL within a few years,^1^ which indicates that the actual safety level of the sound pressure is still under investigation. Because the vestibular examination is often performed in the elderly, the safety level of the acoustic stimulation of VEMPs should be paid attention to, especially the increased vulnerability to loud sounds in aged people who may have a high susceptibility to noise exposure due to various factors. Studies evaluating DPOAE levels and PTA thresholds after VEMP testing in elderly subjects are also required to determine the safety level for the patient population with vestibular disorders. The use of gVEMP (galvanic VEMP) instead of cVEMP or oVEMP in some cases should be considered because galvanic stimulation does not involve the risk of acoustic trauma.

## 4 CONCLUSION

Vestibular evoked myogenic potential sound stimulation can cause noise‐induced hearing loss in some cases with high sensitivity to noise exposure or by exceeding individual cochlear safety thresholds. It is necessary for healthcare providers to understand the risk of hearing loss with VEMP sound stimulation and to explain this to patients in advance. To prevent acoustic trauma, it is necessary to have the patient listen to the stimulus sound before starting the test, to make the patient take a break during the test, and to reduce the number of stimuli and stimulus sound pressure levels. A hearing threshold check after VEMP testing should be considered to make sure there are no hearing problems.

## CONFLICTS OF INTEREST

The authors have no conflicts of interest to disclose.

## AUTHOR CONTRIBUTIONS

All authors contributed to the writing of the manuscript. S. A. and T. K. equally contributed to the study. All authors conceived this study and contributed to the writing of the manuscript.

## ETHICAL APPROVAL

This study was approved by the regional ethical standards committee in the Faculty of Medicine at the University of Tokyo (2487‐(13)).

## CONSENT

The authors have confirmed during submission that patient consent has been signed and collected in accordance with the journal’s patient consent policy.

## Data Availability

Data sharing is not applicable to this article as no datasets were generated or analyzed during the current study.

## References

[1] Portnuff CDFF , Kleindienst S , Bogle JM . Safe use of acoustic vestibular‐evoked myogenic potential stimuli: protocol and patient‐specific considerations. J Am Acad Audiol. 2017;28:708‐717. 28906242 10.3766/jaaa.16071

[2] Mattingly JK , Portnuff CDF , Hondorp BM , Cass SP . Sudden bilateral hearing loss after cervical and ocular vestibular evoked myogenic potentials. Otol Neurotol. 2015;36:961‐964. 25853612 10.1097/MAO.0000000000000764

[3] Stahl MC , Otteson T . Systematic review on vestibular symptoms in patients with enlarged vestibular aqueducts. Laryngoscope. 2021. 10.1002/lary.29819 34397103

[4] Krause E , Mayerhofer A , Gürkov R , et al. Effects of acoustic stimuli used for vestibular evoked myogenic potential studies on the cochlear function. Otol Neurotol. 2013;34:1186‐1192. 23921920 10.1097/MAO.0b013e31829ce7b4

[5] Strömberg AK , Olofsson Å , Westin M , Duan M , Stenfelt S . Changes in cochlear function related to acoustic stimulation of cervical vestibular evoked myogenic potential stimulation. Hear Res. 2016;340:43‐49. 26724755 10.1016/j.heares.2015.12.022

[6] Singh NK , Keloth NK , Sinha S . Is there a safe level for recording vestibular evoked myogenic potential? Evidence from cochlear and hearing function tests. Ear Hear. 2019;40:493‐500. 30148803 10.1097/AUD.0000000000000646

[7] Papathanasiou ES , Murofushi T , Akin FW , Colebatch JG . International guidelines for the clinical application of cervical vestibular evoked myogenic potentials: an expert consensus report. Clin Neurophysiol. 2014;125:658‐666. 24513390 10.1016/j.clinph.2013.11.042

